# Ivermectin Toxicity in Humans and Animals: Clinical Spectrum, Mechanisms, and Management

**DOI:** 10.1002/jat.70166

**Published:** 2026-03-16

**Authors:** Serkan Yilmaz, Bayram Göktaş, İlker Ateş, Mustafa Çelik

**Affiliations:** ^1^ Faculty of Nursing Ankara University Ankara Turkey; ^2^ Haymana Vocational School Ankara University Ankara Turkey; ^3^ Faculty of Pharmacy, Department of Pharmaceutical Toxicology Ankara University Ankara Turkey; ^4^ Faculty of Medicine, Department of Medical Biology Kahramanmaras Sutcu İmam University Kahramanmaras Turkey

**Keywords:** animal, human, ivermectin, toxicity

## Abstract

Ivermectin is a widely used macrocyclic lactone with established efficacy against a broad range of parasitic infections in humans and animals and a long‐standing reputation for clinical safety. However, increasing evidence indicates that ivermectin can produce clinically relevant toxicity under specific conditions, particularly involving the central nervous system. This review integrates findings from controlled human trials, pharmacovigilance data, clinical case reports, experimental animal studies, and environmental investigations to comprehensively characterize the toxicological profile of ivermectin. Early randomized, placebo‐controlled studies in healthy volunteers demonstrated that ivermectin is generally well tolerated, even at doses substantially exceeding approved therapeutic levels, with predominantly mild and transient adverse events and no significant neurological toxicity under controlled conditions. In contrast, post‐marketing surveillance and real‐world clinical reports have identified rare but severe neurotoxic events, including encephalopathy, seizures, coma, and death, occurring after supratherapeutic exposure and, in susceptible individuals, even at standard therapeutic doses. Converging human and animal evidence highlights impairment or saturation of blood–brain barrier protection, particularly dysfunction of P‐glycoprotein (ABCB1/MDR1)–mediated efflux, as a central determinant of ivermectin neurotoxicity. Animal studies further demonstrate marked species‐, breed‐, age‐, dose‐, and route‐dependent susceptibility, with neonatal animals, genetically predisposed dog breeds, and models exposed to transporter inhibition or repeated high‐dose regimens showing pronounced vulnerability. While neurotoxicity is often functional and reversible at lower exposures, high or cumulative dosing can lead to structural neuropathology and multi‐organ injury. The COVID‐19 pandemic amplified these risks through widespread off‐label use, especially of veterinary formulations, resulting in a substantial increase in toxic exposures without demonstrated clinical benefit. Overall, ivermectin toxicity emerges as a predictable consequence of interactions between pharmacokinetics, transporter biology, exposure patterns, and host‐specific factors rather than an inherent contradiction of its therapeutic value.

## Abstract 2

This review synthesizes human and animal evidence on ivermectin toxicity, emphasizing neurotoxicity mechanisms and susceptibility factors. While controlled trials confirm a wide therapeutic margin under standard conditions, pharmacovigilance data and case reports demonstrate rare but severe central nervous system toxicity linked to altered blood–brain barrier function and ABCB1/P‐glycoprotein dysfunction. Animal models corroborate transporter‐mediated vulnerability and dose–formulation effects. Pandemic‐related off‐label and veterinary misuse further highlighted exposure‐dependent risks.

## 1 Introduction

Ivermectin is a broad‐spectrum macrocyclic lactone that has been extensively used for decades in both human and veterinary medicine for the treatment and prevention of parasitic infections. The remarkable clinical impact of ivermectin in the control of neglected tropical diseases was internationally recognized with the 2015 Nobel Prize in Physiology or Medicine awarded to William C. Campbell and Satoshi Ōmura. Their discovery and development of avermectins revolutionized the treatment of parasitic infections such as onchocerciasis and lymphatic filariasis, dramatically reducing disease burden in endemic regions (Campbell et al. 1983). This historical context underscores that ivermectin was originally introduced into human medicine based on its profound therapeutic benefit, which contrasts with the toxicity concerns discussed in the present review.

Its antiparasitic efficacy is primarily attributed to potentiation of glutamate‐gated and γ‐aminobutyric acid (GABA, a Cys‐loop receptor)–mediated chloride conductance in invertebrates, leading to neuromuscular paralysis and parasite death. In mammals, ivermectin is generally considered to have a wide therapeutic margin, largely because its penetration into the central nervous system (CNS) is limited by the blood–brain barrier (BBB) and active efflux mediated by P‐glycoprotein (ABCB1). Acting as an active efflux pump, P‐gp transports lipophilic xenobiotics such as ivermectin from endothelial cells back into the bloodstream, thereby restricting drug accumulation within neural tissue and maintaining a favorable safety margin. Experimental evidence demonstrates that loss of ABCB1 function markedly increases brain concentrations of ivermectin and precipitates severe neurotoxicity, highlighting the transporter's critical gatekeeping role in limiting CNS exposure (Borst and Schinkel 2013). In addition to being a P‐gp substrate, ivermectin may influence transporter expression and activity. Cellular studies have shown that ivermectin exposure can induce time‐ and concentration‐dependent increases in Mdr1a/Mdr1b transcripts and enhance functional efflux capacity, suggesting a dynamic interaction between ivermectin and ABCB1 regulation. Such modulation may represent a compensatory mechanism that further limits brain penetration during systemic exposure (Ménez et al. 2012). The clinical relevance of ABCB1‐mediated efflux is particularly evident in species or individuals carrying loss‐of‐function MDR1 mutations. In certain dog breeds, defective P‐gp activity leads to impaired efflux at the BBB, allowing ivermectin to accumulate in the CNS and overstimulate inhibitory neurotransmission pathways, ultimately resulting in neurological toxicity. These findings collectively emphasize that ABCB1/MDR1 activity is a primary pharmacokinetic determinant controlling ivermectin distribution to the brain and modulating its neurotoxic risk profile (Baydan et al. 2024).

The therapeutic safety of ivermectin is now understood to be tightly linked to ABCB1/MDR1‐mediated drug efflux at the BBB, a concept that emerged from the first ABCB1 knockout mouse studies showing dramatically increased brain accumulation of ivermectin and severe neurotoxicity when P‐glycoprotein (P‐gp) function is absent. These experimental models demonstrated that the BBB acts as an active pharmacokinetic barrier rather than a passive diffusion interface, establishing ABCB1 efflux as a critical determinant of CNS drug exposure and safety margins (Borst and Schinkel 2013; Mealey et al. 2023). The translational relevance of this discovery became evident with the identification of naturally occurring MDR1 mutations in dogs, where frame‐shift variants leading to truncated or dysfunctional P‐gp were associated with markedly increased ivermectin sensitivity and life‐threatening neurological toxicity (Mealey et al. 2023). Similarly, molecular studies in Border Collies revealed insertion mutations and polymorphisms in ABCB1 that may impair efflux capacity and contribute to individual variability in ivermectin tolerance (Han et al. 2010). More recently, comparable loss‐of‐function ABCB1 variants have been described in cats, including the ABCB11930_1931del TC deletion, which abolishes P‐gp drug transport and leads to neurological adverse reactions even at therapeutic ivermectin doses (Nürnberger et al. 2022). These cross‐species observations reinforce earlier cellular and pharmacogenetic studies demonstrating that ivermectin acts as a P‐gp substrate whose CNS exposure is tightly regulated by transporter expression and activity (Ménez et al. 2012; Baydan et al. 2024).

Taken together, evidence from ABCB1 knockout mice, canine and feline MDR1 mutations, and mechanistic transporter studies establishes ABCB1‐mediated efflux at the BBB as a fundamental safeguard against ivermectin‐induced neurotoxicity and highlights pharmacogenetic variation as a key determinant of therapeutic safety across species.

Nevertheless, accumulating evidence indicates that ivermectin toxicity is a dose‐, formulation‐, route‐, and host‐dependent phenomenon, with clinically significant adverse effects occurring under specific conditions in both humans and animals (Chandler 2018; Baudou et al. 2020; Robalino et al. 2025).

**FIGURE 1:**
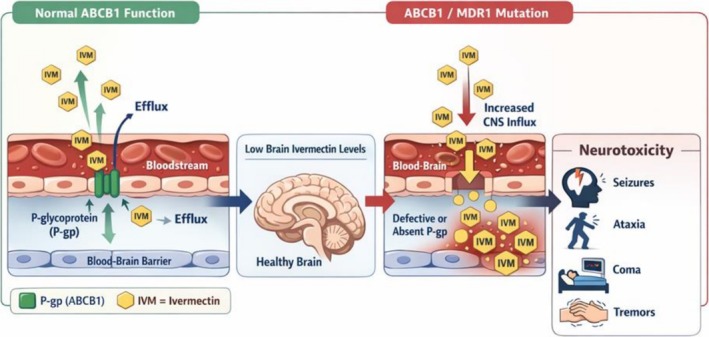
Mechanistic framework of ivermectin neurotoxicity: blood–brain barrier transport, exposure patterns, and clinical outcomes.

Controlled clinical studies in healthy adult volunteers have demonstrated that ivermectin is generally well tolerated even at doses substantially exceeding approved therapeutic levels, with adverse effects typically limited to mild and transient neurological or gastrointestinal symptoms. However, real‐world clinical observations increasingly challenge the assumption of uniform safety, as serious neurotoxic events—including encephalopathy, seizures, ataxia, coma, and, in rare cases, death—have been documented following both therapeutic and supratherapeutic exposure. Importantly, these events often occur independently of dose alone and are strongly influenced by individual susceptibility factors, including impaired BBB integrity, drug–drug interactions, misuse of veterinary formulations, and genetic dysfunction of ABCB1‐mediated efflux. Definitive human evidence has established that loss‐of‐function mutations in ABCB1 can precipitate profound ivermectin neurotoxicity even at standard doses, underscoring the critical role of transporter‐mediated neuroprotection (Baudou et al. 2020; Temple et al. 2021; Donfo‐Azafack et al. 2023) (Figure 1).

The misuse of ivermectin during the COVID‐19 pandemic has further amplified toxicological concerns, with poison center surveillance and clinical case series reporting sharp increases in ivermectin‐related exposures, hospitalizations, and intensive care admissions. These cases reveal a consistent toxidrome dominated by CNS depression, delirium‐like neuropsychiatric manifestations, and neuromuscular dysfunction, particularly following ingestion of veterinary formulations or repeated high‐dose regimens lacking therapeutic justification. The rationale for considering ivermectin as a potential therapeutic option for COVID‐19 largely originated from early in vitro findings demonstrating inhibition of SARS‐CoV‐2 replication. A pivotal cell‐culture study reported that a single exposure of infected Vero‐hSLAM cells to ivermectin produced an approximately 5000‐fold reduction in viral RNA within 48 h, which rapidly stimulated interest in drug repurposing and off‐label clinical use. Mechanistically, the proposed antiviral activity has been linked to disruption of importin‐α/β1‐mediated nuclear transport and interference with host–virus interactions that may limit viral replication and inflammatory signaling. However, the translational relevance of these in vitro observations remains controversial. Subsequent clinical and pharmacokinetic investigations have highlighted that the concentrations required to achieve antiviral effects in cell culture may exceed those attainable with standard human dosing, contributing to inconsistent clinical outcomes and ongoing debate regarding therapeutic efficacy. Consistent with this uncertainty, systematic evaluations of randomized trials have concluded that the available evidence remains inconclusive and of variable certainty, underscoring the need for rigorous critical appraisal when interpreting early laboratory triggers for clinical adoption. (Caly et al. 2020; Popp et al. 2021; Temple et al. 2021; Low et al. 2022; Buonfrate et al. 2022; Kirti et al. 2023).

Animal studies provide convergent and mechanistic support for these clinical observations, demonstrating that ivermectin toxicity is characterized predominantly by neurotoxicity across multiple species, with marked interspecies, age‐dependent, and genetic variability. Neonatal animals and genetically susceptible dog breeds exhibit extreme sensitivity due to immature or defective BBB efflux mechanisms, while high‐dose or repeated exposure in rodents induces oxidative stress, neurochemical disruption, hepatic and renal injury, developmental toxicity, and histopathological brain lesions. Experimental models further show that pharmacological inhibition of P‐glycoprotein dramatically amplifies ivermectin‐induced genotoxicity and teratogenicity, converting an otherwise relatively safe compound into a potent developmental toxicant. Beyond acute toxicity, chronic and sublethal exposure has been shown to induce genetic instability, behavioral impairment, and carcinogenic‐like effects in nontarget organisms, raising concerns about long‐term biological consequences (Hopper et al. 2002; Dadarkar et al. 2007; Dey et al. 2017).

In parallel, environmental studies demonstrate that ivermectin's lipophilicity, persistence, and preferential excretion in animal dung result in sustained environmental contamination, with pronounced toxicity to aquatic invertebrates and measurable ecological disruption. These findings expand the toxicological relevance of ivermectin beyond clinical and veterinary contexts, positioning it within a broader One Health framework that links human health, animal health, and environmental integrity.

Collectively, the available evidence indicates that ivermectin toxicity cannot be adequately characterized by therapeutic dose thresholds alone, but instead reflects a complex interplay of pharmacokinetics, transporter biology, exposure patterns, and host susceptibility. Understanding this complexity is essential for accurate risk assessment, rational clinical use, regulatory decision‐making, and prevention of avoidable adverse outcomes. The present study builds upon this body of evidence by examining ivermectin toxicity in humans and animals through an integrated toxicological perspective, with particular emphasis on neurotoxicity, genetic susceptibility, and dose–exposure relationships.

## 2 Molecular Pharmacology and Target Selectivity

Cys‐loop receptors belong to the pentameric ligand‐gated ion channel (pLGIC) superfamily and mediate rapid synaptic transmission in both vertebrates and invertebrates by converting neurotransmitter binding into ion flux across neuronal membranes. Structurally, these receptors are composed of five homologous subunits arranged around a central pore, each containing a large extracellular ligand‐binding domain, four transmembrane helices (M1–M4), and a variable intracellular loop that regulates trafficking and signaling (Nys et al. 2013; Nicholl et al. 2017). The conserved disulfide‐bonded “Cys‐loop” motif located within the extracellular domain couples agonist binding to channel gating, enabling transitions between closed, open, and desensitized conformational states that underlie fast excitatory or inhibitory neurotransmission (Hernando et al. 2023). In vertebrates, major Cys‐loop receptor families include nicotinic acetylcholine, 5‐HT3 serotonin, GABA(A), and glycine receptors, whereas invertebrates possess a broader repertoire that includes glutamate‐gated chloride channels and unique ligand‐specific variants, making them important pharmacological targets for insecticides and anthelmintics (Hernando et al. 2023; Lynagh and Lynch 2012). Notably, ivermectin exerts its antiparasitic effects by binding at intersubunit sites within the transmembrane domain of invertebrate Cys‐loop receptors, stabilizing open channel conformations and increasing chloride conductance, while mammalian receptors exhibit lower sensitivity, contributing to therapeutic selectivity 8 Lynagh and Lynch 2012). Electrophysiological studies in nematodes further demonstrate that evolutionary diversification of Cys‐loop receptor subtypes results in distinct pharmacological profiles, including both inhibitory and excitatory GABA‐gated channels, emphasizing their structural plasticity and drug‐target relevance across species.

High‐resolution analyses by Hibbs and Gouaux (2011) have recently clarified the molecular basis of ivermectin binding, demonstrating its interaction with the homomeric α‐GluClR in *C. elegans* . Ivermectin binds within the transmembrane domain at the interface between adjacent receptor subunits, where it stabilizes an open‐channel conformation through allosteric modulation rather than classical orthosteric agonism. Structural studies demonstrate that the ligand wedges between M1 and M3 helices, inducing conformational rearrangements that propagate toward the M2 pore‐lining region and enhance chloride conductance. This mechanism differs from endogenous neurotransmitter activation, as ivermectin produces slow‐onset and long‐lasting channel opening, which can prolong inhibitory signaling and potentially contribute to neurotoxicity when CNS exposure increases. Although vertebrate GABA(A) receptors are less sensitive than invertebrate GluCl channels, micromolar ivermectin concentrations can directly activate or potentiate GABAergic currents (Hibbs and Gouaux 2011; Lynagh and Lynch 2012).

## 3 Materials and Methods

### 3.1 Literature Search Strategy

A systematic literature search was conducted to identify peer‐reviewed studies addressing ivermectin toxicity, pharmacodynamics, and transporter‐mediated safety mechanisms in humans and animals. Four electronic databases were searched independently to ensure comprehensive coverage of biomedical, pharmacological, and veterinary research: PubMed/MEDLINE, Web of Science, Scopus, and Google Scholar. Searches were performed using predefined keyword combinations and Boolean operators (AND/OR) to maximize transparency and reproducibility.

The search syntax included the following primary keyword clusters:

- *ivermectin* AND (“toxicity” OR “neurotoxicity” OR “adverse effects” OR “poisoning” OR “safety”);
- *ivermectin* AND (“Cys‐loop receptor” OR “GABA(A)” OR “ligand‐gated ion channel”);
- *ivermectin* AND (“ABCB1” OR “MDR1” OR “P‐glycoprotein” OR “drug efflux transporter”);
- transporter‐related pharmacogenetic terms including (“mutation” OR “polymorphism” OR “knockout” OR “deficiency”) combined with species identifiers (“mouse” OR “mice” OR “dog” OR “canine” OR “cat” OR “feline” OR “human”). These transporter‐focused keywords were specifically included to capture studies addressing ABCB1/MDR1‐associated ivermectin sensitivity and blood–brain barrier drug efflux mechanisms.

Controlled vocabulary (e.g., MeSH terms in PubMed) and free‐text searches were applied where available. Reference lists of eligible articles were manually screened to identify additional relevant studies not retrieved by database queries. Searches were restricted to peer‐reviewed publications written in English, and duplicate records were removed prior to screening. Titles and abstracts were evaluated for relevance to ivermectin toxicity, pharmacology, or transporter‐mediated safety, followed by full‐text assessment according to predefined inclusion criteria described in the Study Selection Process section.

### 3.2 Eligibility Criteria

Primary research articles reporting toxicological outcomes of ivermectin in humans or animals; clinical studies, case reports/series, experimental animal studies, and in vivo toxicological investigations; and only articles published in English were included.

Focused solely on therapeutic efficacy without reporting toxic or adverse effects, review articles, editorials, commentaries, or conference abstracts addressed only antiparasitic efficacy or antiviral hypotheses without toxicological endpoints and were in vitro–only studies without in vivo relevance.

### 3.3 Study Selection Process

The initial database search yielded 3170 records. After removal of duplicates, titles and abstracts were screened for relevance to ivermectin toxicity. Articles that clearly did not meet the inclusion criteria were excluded at this stage. The remaining studies underwent full‐text assessment for eligibility. Following full‐text review, studies were excluded if toxicological data were insufficient or if the outcomes did not directly address ivermectin‐induced adverse effects. Ultimately, 39 studies met the inclusion criteria and were included in the qualitative synthesis.

### 3.4 Data Extraction and Classification

For each included study, data were extracted independently and systematically, including study type and design; species (human or animal); exposure route, dose, and formulation of ivermectin; reported toxicological endpoints (e.g., neurotoxicity, hepatotoxicity, genotoxicity, developmental toxicity); and key mechanistic findings and susceptibility factors (e.g., ABCB1/P‐glycoprotein involvement). The included studies were categorized into mechanism (*n* = 15), human toxicity studies (*n* = 11), and animal toxicity studies (*n* = 13) to allow structured comparison across species and exposure contexts.

### 3.5 Methodological Framework

This study follows the principles of the Preferred Reporting Items for Systematic Reviews and Meta‐Analyses (PRISMA) guidelines for transparent reporting of literature identification, screening, and inclusion. Given the heterogeneity of study designs, exposure scenarios, and outcome measures, a qualitative synthesis approach was adopted rather than meta‐analysis.

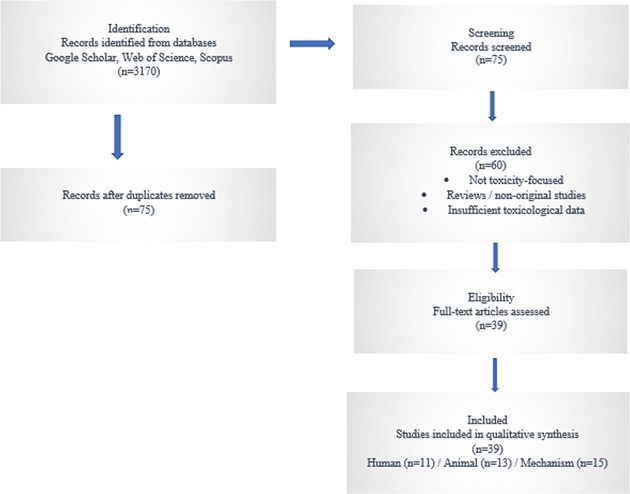

## 4 Human Studies

Early human investigations into ivermectin safety were conducted under rigorously controlled clinical conditions and collectively established the drug's reputation for a wide therapeutic index (Table 1). The pivotal randomized, double‐blind, placebo‐controlled dose‐escalation study by Guzzo et al. (2002) evaluated the safety, tolerability, and pharmacokinetics of ivermectin administered at doses substantially exceeding approved therapeutic levels. Healthy adult volunteers received single or repeated oral doses up to 120 mg—approximately tenfold higher than the highest FDA‐approved dose on a weight‐adjusted basis. Across all dosing regimens, ivermectin was generally well tolerated, with adverse events predominantly mild and transient, consisting mainly of headache, dizziness, and gastrointestinal symptoms, and without clinically significant neurological toxicity.

**TABLE 1:** Summary of human studies on ivermectin toxicity.

Study (year)	Studied population	Formulation	Dose/exposure	Study type	Target system/tissue	Key toxicological findings	Mechanistic insight/line reference
Guzzo et al. (2002)	Healthy adult volunteers	Human oral tablets	30–120 mg single/repeated dosing (≈10 × FDA dose)	Randomized double‐blind clinical trial	CNS; plasma PK	Generally well tolerated; no significant neurotoxicity	Therapeutic‐range exposure with intact ABCB1 efflux
Chandler (2018)	Global pharmacovigilance cases	Human therapeutic formulations	Standard therapeutic dosing	WHO VigiBase analysis	CNS	Rare severe neurotoxicity independent of Loa loa	Post‐marketing evidence of CNS penetration
Baudou et al. (2020)	13‐year‐old male	Human oral tablet	0.23 mg/kg single prophylactic dose	Case report with genetic analysis	CNS; BBB	Severe neurotoxicity despite therapeutic dosing	ABCB1 LoF mutations: p.Arg794Ter + p.Ile1018ThrfsTer8
Morales et al. (2021)	Elderly woman	Veterinary equine formulation	≈227.5 mg oral ingestion (2 × 113.75 mg ampules)	Case report	CNS	Acute encephalopathy; reversible	Veterinary formulation → supratherapeutic exposure
Temple et al. (2021)	Poison center cases (USA)	1.87% equine paste; 1% livestock solution	6.8–125 mg single doses; repeated 21 mg tablet use	Retrospective poison center analysis	CNS; systemic	Increased toxicity during COVID‐19; ICU admissions	Dose magnitude + formulation dependent risk
Hoang et al. (2022)	37 adult patients	Human tablets + veterinary paste/solution	Mean 95.1 mg/day (veterinary); 13.5 mg/day chronic tablets	Retrospective clinical cohort	CNS; GI; musculoskeletal	Neurotoxicity predominant; veterinary formulations more severe	P‐gp inhibitor co‐medication amplified toxicity
Dy and Juangco (2023)	52‐year‐old male	Human tablets	108 mg + 216 mg within 24 h (> 10× cumulative dose)	Detailed case analysis	CNS	Defined ivermectin toxidrome; rapid reversal with charcoal	Efflux saturation → transient ↑ CNS exposure
Reis et al. (2022)	1358 COVID‐19 outpatients	Human tablets	400 μg/kg/day × 3 days	Randomized controlled trial	Clinical outcomes	No clinical benefit; safety similar to placebo	Therapeutic exposure without transporter compromise
Hernandez et al. (2024)	7035 COVID‐19 patients	Human therapeutic formulations	Variable dosing across RCTs	Systematic review and meta‐analysis	Clinical outcomes	No benefit; higher cumulative doses ↑ hospitalization risk	Exposure intensity influences safety profile
Donfo‐Azafack et al. (2023)	19‐year‐old female	Human tablets (3 mg)	≈ 1200 mg intentional overdose (~100× dose)	Case report	CNS	Massive overdose; reversible toxicity	Redistribution‐driven recovery without antidote
Yin et al. (2025)	Adult woman	1% veterinary dermal solution	≈2 g/day dermal exposure × 1 month	Fatal case with toxicokinetics	CNS; systemic	Fatal CNS toxicity after chronic dermal absorption	Prolonged dermal exposure → lethal accumulation

A major strength of the study by Guzzo et al. (2002) was the use of objective neurological and neuro‐ophthalmologic assessments, including quantitative pupillometry, to monitor potential CNS toxicity. Even at escalating single doses ranging from 30 to 120 mg (approximately 333–2000 μg/kg), no evidence of CNS involvement—such as ataxia, altered mental status, or clinically relevant mydriasis—was detected. Pharmacokinetic analyses further demonstrated that systemic exposure increased in an approximately dose‐proportional manner across this range, with both AUC and Cmax rising with increasing dose while tmax (~4 h) and elimination half‐life (~15–20 h) remained relatively stable, consistent with linear kinetics. For example, geometric mean AUC0‐∞ values increased from roughly 1724 ng·h/mL at 30 mg (fasted) to 4547 ng·h/mL at 120 mg, and Cmax rose from about 84.8 to 247.8 ng/mL, supporting a clear dose‐dependent increase in systemic exposure. Repeated dosing on Days 1, 4, and 7 resulted in minimal accumulation (AUC day 7/day 1 ratios of 1.24–1.40), indicating limited drug build‐up under the studied regimen. In addition, a pronounced food effect was observed, with administration alongside a high‐fat meal increasing bioavailability approximately 2.5‐fold compared with the fasted state. Importantly, plasma concentrations remained well below those associated with neurotoxicity in susceptible animal models, supporting a wide margin of safety under controlled clinical conditions.

Despite these reassuring findings from controlled clinical trials, post‐marketing surveillance and real‐world clinical observations have revealed that ivermectin‐associated neurotoxicity, although rare, is a genuine and clinically significant phenomenon. An analysis of serious neurological adverse events reported to VigiBase, the WHO global pharmacovigilance database, by Chandler (2018) identified cases of encephalopathy, seizures, ataxia, depressed consciousness, and coma temporally associated with ivermectin exposure. Symptom onset typically occurred within hours to days following standard therapeutic dosing, highlighting that toxicity may arise even in the absence of overt overdose.

Crucially, Chandler (2018) demonstrated that several reports provided supportive evidence of causality, including positive dechallenge and rechallenge, as well as direct detection of ivermectin in brain tissue in one fatal case. Historically, severe post‐ivermectin encephalopathy was primarily reported in individuals co‐infected with Loa loa, a filarial nematode endemic to West and Central Africa. In these settings, patients with high circulating microfilarial loads may develop intense inflammatory responses after ivermectin‐induced parasite killing, leading to endothelial activation, microvascular injury, and disruption of the BBB, which has been proposed as a key mechanism underlying neurological deterioration during mass drug‐administration programs. Consequently, screening strategies and post‐treatment monitoring were implemented in endemic regions to reduce the risk of severe adverse events. However, Chandler's analysis identified serious neurological reactions even in the absence of documented loiasis, indicating that neurotoxicity cannot be explained solely by Loa loa co‐infection. Instead, attention has shifted toward host‐specific susceptibility factors. Under physiological conditions, ivermectin penetration into the CNS is limited by the integrity of the BBB, where endothelial tight junctions and P‐glycoprotein (mdr‐1) efflux transporters actively restrict drug entry. Disruption of this barrier—whether through systemic inflammation, genetic variability affecting transporter function, or pharmacokinetic interactions that inhibit efflux mechanisms—may allow increased CNS exposure and contribute to rare but severe neurotoxic events.

Definitive mechanistic insight into these susceptibility factors was provided by the pediatric case reported by Baudou et al. (2020). A 13‐year‐old boy developed severe neurotoxicity—including coma and focal neurological deficits—after a single oral prophylactic dose of ivermectin (0.23 mg/kg), demonstrating that toxicity may occur even within the conventional therapeutic range. Genetic analysis identified compound heterozygous ABCB1 nonsense mutations—c.2380C → T [p.(Arg794Ter)] in exon 20 and c.3053_3056delITTGA [p.(Ile1018ThrfsTer8)] in exon 25—each generating truncated P‐glycoprotein lacking functional nucleotide‐binding domains required for ATP‐dependent efflux at the BBB. Loss of transporter activity resulted in impaired drug extrusion from endothelial cells of the barrier and excessive CNS exposure, explaining the unexpected severity of toxicity despite therapeutic dosing. Comparable mechanisms have long been recognized in veterinary medicine, where inherited ABCB1 loss‐of‐function variants—such as the nt230(del4) deletion in collie‐type dogs and the nt1930(del2) deletion reported in cats—reduce P‐glycoprotein activity and permit ivermectin accumulation in neural tissue. The patient recovered fully with supportive care alone, and this case provided the first clear human evidence that ABCB1 dysfunction represents a key genetic determinant of ivermectin neurotoxicity and may account for rare severe reactions observed despite the drug's otherwise favorable safety profile.

The clinical significance of these vulnerability factors became particularly evident during the COVID‐19 pandemic, when ivermectin use expanded beyond approved indications. Morales et al. (2021) described acute encephalopathy in a 70‐year‐old woman following accidental ingestion of a veterinary ivermectin preparation intended for horses, obtained over the counter for presumed COVID‐19 treatment. The patient ingested two ampules of an equine ivermectin formulation, each containing 113.75 mg of ivermectin, resulting in an estimated exposure far exceeding recommended human dosing. Approximately 5 h after ingestion, she developed rapidly progressive altered mental status without focal neurological deficits, while brain CT imaging remained unremarkable. Supportive management alone led to gradual neurological recovery within 48 h, highlighting the neurotoxic risk associated with high‐dose exposure and misuse of veterinary formulations.

Population‐level evidence of this emerging public health issue was provided by poison center–based analyses. Temple et al. (2021) documented a marked increase in ivermectin‐related toxic exposures reported to the Oregon Poison Center during surges in off‐label COVID‐19 use. Among the reported cases, hospitalization—including intensive care admission—was associated with large single or repeated doses, particularly ingestion of veterinary products such as 1.87% paste (6.8–125 mg) and 1% solution formulations (20–50 mg), as well as repeated dosing with human tablets at 21 mg per dose twice weekly for prevention. Six patients required hospitalization, and four were treated in intensive care units, with clinical manifestations dominated by neurotoxicity, including confusion, ataxia, seizures, and hypotension. Veterinary formulations were disproportionately represented among severe cases, underscoring the role of dose magnitude and formulation in toxicity risk.

The most comprehensive clinical characterization of ivermectin toxicosis in humans was subsequently provided by Hoang et al. (2022) in a retrospective poison center analysis. Among 37 patients, neurotoxicity emerged as the most frequent manifestation, encompassing altered mental status, ataxia, visual disturbances, tremors, seizures, and hallucinations. Patients who ingested veterinary formulations—primarily 1.87% equine ivermectin paste and 1% liquid solution products commonly used for livestock—consumed substantially higher daily doses (mean ≈95.1 mg/day) and experienced more severe and rapid‐onset toxicity than those taking prescription tablets. In contrast, chronic users developed milder toxicity despite prolonged exposure to doses closer to approved antiparasitic regimens (median daily dose ≈13.5 mg over several weeks). These findings highlight the importance of formulation type and dosing pattern in determining the clinical severity of ivermectin toxicity.

Importantly, Hoang et al. (2022) also identified potential amplification of neurotoxicity in patients concurrently using P‐glycoprotein inhibitors, including macrolide antibiotics, proton pump inhibitors, and certain β‐blockers, supporting transporter‐mediated mechanisms of increased CNS exposure. Their analysis further distinguished acute from chronic ivermectin toxicity patterns. Acute toxicity—typically resulting from large single ingestions or short‐term supratherapeutic dosing of veterinary formulations—was characterized by rapid onset of neurotoxicity, including severe altered mental status, seizures, and visual disturbances. In contrast, chronic toxicity developed in patients taking smaller daily doses (approximately 13.5 mg/day) over several weeks and was generally associated with milder neurological and gastrointestinal symptoms without severe encephalopathy. These observations reinforce the concept that ivermectin toxicity is not solely dose‐dependent but reflects a complex interaction between formulation type, exposure pattern (acute versus chronic), individual susceptibility, and pharmacokinetic modifiers that alter P‐glycoprotein–mediated efflux at the BBB.

Dy and Juangco (2023) further refined the clinical characterization of ivermectin toxicity by describing a 52‐year‐old man who developed acute neurotoxicity after self‐administering extremely high cumulative doses of ivermectin for presumed COVID‐19 treatment. The patient initially ingested 108 mg followed by an additional 216 mg within 24 h, corresponding to an estimated cumulative exposure of approximately 4 mg/kg—more than 10 times the recommended therapeutic dose—after which he developed decreased sensorium, agitation, tremulousness, gait instability, and complex visual hallucinations. Neuroimaging remained unremarkable, while electroencephalography demonstrated diffuse cerebral slowing consistent with toxic‐metabolic encephalopathy. Although administration of activated charcoal was temporally associated with rapid clinical improvement and resolution of symptoms within 24–48 h, spontaneous recovery cannot be excluded, given ivermectin's pharmacokinetic profile and the absence of a specific antidote. The authors proposed a recognizable ivermectin toxidrome characterized by CNS depression and delirium‐like features following supratherapeutic exposure. Importantly, the duration and severity of intoxication are likely influenced by the functional integrity of ABCB1/MDR1‐mediated efflux at the BBB: Intact transporter activity limits central accumulation and facilitates recovery, whereas impaired or saturated efflux may prolong neurotoxicity by allowing sustained CNS exposure.

In parallel with the accumulation of toxicity data, high‐quality randomized controlled trials failed to demonstrate any therapeutic benefit of ivermectin in COVID‐19. The ivermectin arm of the TOGETHER trial reported by Reis et al. (2022) showed no reduction in hospitalization, mortality, or other clinically meaningful outcomes compared with placebo. These findings were corroborated by a comprehensive systematic review and meta‐analysis by Hernandez et al. (2024), which demonstrated no benefit across primary or secondary outcomes and suggested increased hospitalization risk with higher cumulative ivermectin doses.

Finally, extreme exposure scenarios have delineated the outer limits of ivermectin toxicity in humans. Donfo‐Azafack et al. (2023) described a 19‐year‐old woman who intentionally ingested approximately 400 tablets of ivermectin 3 mg (estimated ~1200 mg, nearly 100‐fold the recommended therapeutic dose), resulting in CNS depression characterized by somnolence, kinetic ataxia, hyperreflexia, mydriasis, and visual disturbances. Despite this massive exposure, vital signs and laboratory parameters remained largely stable, and management consisted primarily of supportive care, hydration, and symptomatic treatment, with gradual neurological recovery over several days, illustrating the reversibility of toxicity even at extreme oral doses. In contrast, Yin et al. (2025) reported the first fatal case of transdermal ivermectin poisoning with quantified plasma concentrations. A woman in her early 40s repeatedly applied approximately 200 mL of a 1% veterinary ivermectin solution to ulcerated skin under occlusion for 1 month, leading to progressive gastrointestinal symptoms followed by coma, diffuse cerebral edema, and intracranial hypertension; toxicological analysis confirmed a plasma concentration of 27 ng/mL, far exceeding typical dermal exposure levels. Imaging demonstrated severe cerebral edema with loss of sulcal architecture and subsequent cerebral circulatory arrest, and the patient ultimately died despite intensive care, hemoperfusion, and osmotherapy. Together, these cases emphasize that toxicity risk is strongly influenced not only by total dose but also by route of exposure, formulation, duration of contact, and integrity of biological barriers such as the skin and BBB.

## 5 Pharmacological Modulation of Ivermectin Toxicity: Transporter, Metabolic, and Pharmacodynamic Mechanisms

Accumulating evidence indicates that ivermectin toxicity is not solely dose‐dependent but can be substantially modified by pharmacological factors that alter drug disposition, BBB permeability, and neuronal susceptibility. The most extensively characterized mechanism involves modulation of ATP‐binding cassette transporters, particularly P‐glycoprotein (ABCB1), which actively restricts ivermectin penetration into the CNS (Borst and Schinkel 2013; Ménez et al. 2012; Baudou et al. 2020; Mealey et al. 2023). Concomitant administration of P‐glycoprotein inhibitors—including macrolide antibiotics and experimental agents such as verapamil—has been associated with increased systemic exposure and enhanced neurotoxicity in both clinical and experimental settings (Hoang et al. 2022; El‐Ashmawy et al. 2011).

Metabolic modulation represents a second critical pathway. Ivermectin undergoes extensive hepatic biotransformation, and nonlinear pharmacokinetics have been observed at supratherapeutic doses, suggesting saturation of metabolic and efflux systems (Guzzo et al. 2002; Marjanović et al. 2024). Experimental studies demonstrate dose‐dependent oxidative stress, hepatocellular injury, and secondary renal and reproductive toxicity, indicating that pharmacological interference with metabolic clearance may amplify systemic toxicity (Dadarkar et al. 2007; Almawla and Al‐Baggou 2023; Khalifa et al. 2025).

Beyond pharmacokinetic mechanisms, pharmacodynamic modulation has also been described. Antioxidant therapies and receptor‐targeted interventions partially attenuated ivermectin‐induced oxidative and neurochemical alterations in preclinical models, supporting the concept that downstream signaling pathways influence toxicity severity independently of administered dose (El‐Ashmawy et al. 2011; Almawla and Al‐Baggou 2023; Khalifa et al. 2025).

## 6 Animal Studies

Animal studies have provided foundational insight into the neurotoxic and systemic effects of ivermectin, particularly by elucidating species‐specific susceptibility, dose–response relationships, and the mechanistic role of BBB protection (Table 2). Pioneering work by Schinkel and colleagues using Abcb1 (Mdr1a) knockout mice demonstrated that disruption of P‐glycoprotein–mediated efflux at the BBB results in dramatically increased brain penetration of ivermectin and approximately 100‐fold greater sensitivity to neurotoxicity compared with wild‐type animals, establishing transporter function as a primary determinant of CNS safety. Complementary evidence of developmental vulnerability was reported by Sanford et al. (1988), who described severe ivermectin toxicosis in neonatal piglets following whole‐herd subcutaneous administration. Clinical signs were confined to piglets aged 2–7 days and consisted predominantly of acute CNS manifestations, including lethargy, tremors, ataxia, incoordination, vomiting, and lateral recumbency. Despite the absence of gross or histopathological brain lesions, markedly elevated ivermectin concentrations were detected in hepatic tissue, exceeding adult reference levels by more than an order of magnitude. Rather than reflecting a structurally “incomplete” barrier, the authors attributed toxicity to dosing inaccuracies combined with reduced functional efflux capacity and developmental immaturity of BBB transport systems, highlighting age‐dependent physiology as a critical determinant of ivermectin neurotoxicity.

**TABLE 2:** Summary of animal studies on ivermectin toxicity.

Study (year)	Species/model	Dose/exposure	Study type	Target tissue(s)	Key toxicological findings
Sanford et al. (1988)	Neonatal piglets	Subcutaneous overdose	Field outbreak report	CNS; liver	Severe age‐dependent neurotoxicity; high hepatic ivermectin levels; immature BBB implicated
Hopper et al. (2002)	Adult Collie dogs	≥ 200–400 μg/kg (oral/SC)	Retrospective veterinary study	CNS	Severe, prolonged neurotoxicity; breed‐specific susceptibility due to MDR1 deficiency
Dadarkar et al. (2007)	Rats	5–50 mg/kg SC	Acute toxicity study (LD)	CNS; liver	Dose‐dependent CNS depression; mild hepatic injury; functional neurotoxicity predominates
El‐Ashmawy et al. (2011)	Pregnant rats + fetuses	Ivermectin ± verapamil	Reproductive and genotoxicity study	Fetal tissues; placenta	P‐gp inhibition converted ivermectin into potent teratogen; placental efflux is protective
Domingues et al. (2016)	Adult zebrafish	0.25–25 μg/L (chronic)	Environmental toxicity study	CNS; oxidative stress pathways	Behavioral neurotoxicity at sublethal doses; impaired feeding and growth
Dey et al. (2017)	Doberman pups	50–1600× canine dose	Veterinary case series	CNS; muscle	Profound neurotoxicity; prolonged recovery; formulation misuse critical
Udhavrao et al. (2017)	Calf	≈13 × recommended oral dose	Veterinary case report	CNS; respiratory system	Severe neuro‐respiratory signs; complete recovery with supportive care
Mesa et al. (2017)	Aquatic invertebrates	Dung‐derived ivermectin	Microcosm ecosystem study	Invertebrates; sediment	High nontarget toxicity; persistent environmental exposure
Almawla and Al‐Baggou (2023)	Mice	30–100 mg/kg oral	Acute and subacute toxicity	Brain; liver	Oxidative stress; neurotransmitter imbalance; dose‐dependent neurotoxicity
Shwaish et al. (2024)	Rabbits	33–67 mg/kg SC	Experimental poisoning	CNS (histopathology)	Structural brain damage; limited therapeutic reversal
Marjanović et al. (2024)	Rats	3–24× human antiparasitic dose	Short‐term repeated dosing	Brain; liver; kidney; testes	Nonlinear PK; brain accumulation; multi‐organ injury
Khalifa et al. (2025)	Rats	High‐dose acute exposure	Mechanistic toxicity study	Brain; skin	Oxidative, inflammatory, microRNA‐mediated neurocutaneous toxicity

Breed‐specific susceptibility to ivermectin neurotoxicity was subsequently characterized in dogs, most notably in Collies. In a retrospective clinical analysis, Hopper et al. (2002) reported prolonged and severe neurotoxic courses in adult Collies exposed to doses commonly used off‐label for parasitic infestations. Affected animals developed progressive ataxia, obtundation, hypersalivation, mydriasis, bradycardia, and in severe cases, coma requiring mechanical ventilation. Oral exposure was associated with more rapid onset and greater severity, whereas subcutaneous administration produced delayed but prolonged deterioration. The authors linked this marked susceptibility to impaired P‐glycoprotein (MDR1)–mediated efflux at the BBB, providing seminal evidence that genetic transporter dysfunction profoundly amplifies ivermectin neurotoxicity in mammals.

Quantitative toxicological benchmarks for ivermectin have been established through controlled laboratory studies. Dadarkar et al. (2007) evaluated acute toxicity following single subcutaneous administration in rats and reported LD values ranging from approximately 5–50 mg/kg depending on the analytical method employed. High‐dose exposure produced dose‐dependent CNS depression characterized by lethargy, ataxia, loss of righting reflex, and mortality, while surviving animals recovered within 24 h. Histopathological examination revealed mild hepatic injury but no overt structural brain damage, reinforcing the concept that ivermectin neurotoxicity is predominantly functional rather than structural at acute exposure levels.

The protective role of P‐glycoprotein in limiting fetal and CNS exposure was further demonstrated in reproductive toxicity studies. El‐Ashmawy et al. (2011) showed that ivermectin administration during organogenesis in pregnant rats produced minimal developmental toxicity when given alone. In contrast, co‐administration with the P‐glycoprotein inhibitor verapamil resulted in pronounced teratogenic and genotoxic effects, including fetal malformations, increased resorptions, and chromosomal aberrations. These findings provide compelling experimental evidence that pharmacological inhibition of efflux transporters can convert ivermectin into a potent developmental toxicant.

Beyond mammalian models, aquatic organisms have proven highly sensitive to ivermectin exposure. Chronic exposure studies in adult zebrafish conducted by Domingues et al. (2016) demonstrated significant concentration‐dependent behavioral toxicity following 21‐day exposure to 0.25, 2.5, and 25 μg/L ivermectin. Even the lowest concentration tested (0.25 μg/L) disrupted swimming behavior, causing fish to remain predominantly at the bottom of the aquaria and impairing feeding efficiency, while higher concentrations produced lethargy, weight loss, and mild morphological alterations. Behavioral endpoints were particularly sensitive, with an estimated EC50 of approximately 1.6–5 μg/L for swimming and feeding impairment, whereas acute lethality occurred only at much higher levels (96‐h LC50 ≈ 73.3 μg/L). Biochemical analyses further revealed oxidative stress responses, including inhibition of catalase and glutathione‐S‐transferase activity at 25 μg/L, despite limited mortality. These findings indicate that ivermectin can disrupt CNS–mediated behaviors in aquatic vertebrates at sublethal, environmentally relevant concentrations, with potentially significant ecological consequences.

Severe ivermectin toxicosis resulting from formulation misuse has also been documented in veterinary practice. Dey et al. (2017) reported profound neurotoxicity in Doberman pinscher pups following accidental administration of a cattle ivermectin formulation at doses up to 1600‐fold higher than recommended. Clinical signs included coma, mydriasis, blindness, and neuromuscular dysfunction, accompanied by marked biochemical evidence of systemic stress. Although survival was achieved in some animals with intensive supportive care, neurological recovery was prolonged, underscoring the extreme vulnerability of young animals to dosing errors and inappropriate formulations.

Comparable findings were reported in ruminants, where Udhavrao et al. (2017) described ivermectin toxicity in a 1‐month‐old Deoni calf weighing 39 kg following oral administration of a dewormer bolus containing ivermectin (100 mg) and fenbendazole. The calf developed salivation, tachypnea, tremors, incoordination, and prolonged recumbency consistent with neuro‐respiratory toxicity; clinical signs were attributed primarily to ivermectin overdose, estimated to be approximately 13 times higher than the standard therapeutic dose. Supportive therapy with fluids, anti‐inflammatory medication, antihistamines, and corticosteroids resulted in progressive improvement beginning on Day 2 and complete recovery by Day 4. Rather than implying an intrinsically wide safety margin, this case illustrates that the severity and reversibility of ivermectin toxicosis in livestock are strongly modulated by pharmacokinetic variables—particularly age‐related susceptibility, oral bioavailability, and route‐dependent absorption—which may limit systemic exposure despite high administered doses.

Environmental exposure pathways have further expanded the toxicological profile of ivermectin. Using aquatic microcosm systems, Mesa et al. (2017) demonstrated that ivermectin excreted in cattle dung accumulates in sediment and nontarget organisms at environmentally relevant concentrations. In their experimental design, dung was spiked with 22, 50, 458, and 1150 μg kg^−1^ ivermectin—levels corresponding to residues detected between Days 3 and 29 following cattle treatment—and measurable accumulation in sediment + dung increased over time, reaching approximately 119.4 ng g^−1^ by Day 17 at the highest exposure level. Even intermediate concentrations caused substantial ecological effects, including complete mortality of sensitive aquatic invertebrates such as *Ceriodaphnia dubia* and *Hyalella* at 458–1150 μg kg^−1^, while accumulation in macrophyte roots and snails increased proportionally with exposure. These quantitative findings clarify that ivermectin residues do not merely “rapidly accumulate,” but persist and concentrate within the sediment–dung matrix and associated food webs at levels capable of producing pronounced ecological toxicity.

Experimental studies in rabbits by Shwaish et al. (2024) provided detailed neuropathological confirmation of ivermectin‐induced CNS injury. Following subcutaneous administration, animals developed marked autonomic and neurological disturbances, with histopathological examination revealing leukoencephalomalacia, gliosis, neuronal degeneration, and perivascular edema. Therapeutic interventions yielded limited benefit, emphasizing the narrow safety margin once severe neurotoxicity is established.

More recent rodent studies have highlighted the risk of cumulative organ toxicity associated with repeated high‐dose exposure. Marjanović et al. (2024) showed that 5‐day oral administration of ivermectin at 0.6–4.8 mg/kg (3–24× the standard antiparasitic dose) induced histopathological changes in the liver, kidneys, testes, and brain despite minimal clinical signs. Hepatic intoxication was characterized by increased ALT and γ‐GT together with hepatocellular edema, vacuolar degeneration, and focal necrosis, suggesting metabolic overload and cytochrome P450–mediated injury. Renal lesions, including tubular dilation, intracellular edema, and glomerular sclerosis, likely reflected systemic accumulation and direct tubular toxicity rather than overt renal failure. Testicular damage emerged only at the highest dose and involved degeneration of seminiferous epithelium with reduced spermatogenesis, indicating selective vulnerability of rapidly dividing germ cells. Nonlinear pharmacokinetics at supratherapeutic doses—marked by saturation of metabolic enzymes and efflux transporters—resulted in disproportionate tissue and brain accumulation, providing a mechanistic basis for multisystem toxicity.

At the molecular level, Almawla and Al‐Baggou (2023) and Khalifa et al. (2025) demonstrated that ivermectin toxicity is accompanied by oxidative stress, neurotransmitter dysregulation, inflammatory signaling, and altered expression of P‐glycoprotein and stress‐related microRNAs in brain and peripheral tissues. Partial mitigation with antioxidants or receptor antagonists supports a multifactorial mechanism involving oxidative, neurochemical, and transporter‐mediated pathways.

Collectively, animal studies provide converging evidence that ivermectin toxicity is governed by species‐ and age‐specific susceptibility, genetic and pharmacological modulation of efflux transporters, dose magnitude, formulation, and exposure duration. These findings not only explain the heterogeneity observed in human toxicity reports but also reinforce the translational relevance of animal models in understanding ivermectin‐induced neurotoxicity across biological systems.

## 7 Conclusion

Collectively, evidence from both human and animal studies demonstrates that ivermectin possesses a wide therapeutic margin under controlled conditions, yet exhibits a clinically meaningful potential for neurotoxicity and systemic toxicity under specific circumstances. Early controlled human trials established that ivermectin is generally well tolerated at doses substantially exceeding approved therapeutic levels in healthy adults, with minimal neurological adverse effects observed under regulated clinical settings. However, post‐marketing surveillance, pharmacovigilance analyses, and detailed case reports have unequivocally shown that serious neurotoxicity can occur, even at standard therapeutic doses, in susceptible individuals.

A central and unifying finding across human and animal literature is the critical protective role of BBB integrity and transporter‐mediated efflux, particularly via the ATP‐binding cassette transporter ABCB1, historically referred to as MDR1, which encodes the efflux protein P‐glycoprotein. Although the terms ABCB1, MDR1, and P‐glycoprotein are often used interchangeably, ABCB1 denotes the gene, MDR1 represents its classical pharmacogenetic designation, and P‐glycoprotein refers to the functional transmembrane transporter expressed on endothelial cells of the BBB. Under physiological conditions, P‐glycoprotein actively transports ivermectin from the luminal membrane of brain capillary endothelial cells back into the systemic circulation, thereby limiting passive diffusion across tight junction–sealed paracellular pathways and maintaining low CNS exposure. Experimental knockout models, pharmacological inhibition studies, and naturally occurring loss‐of‐function mutations consistently demonstrate that disruption or saturation of ABCB1/P‐glycoprotein–mediated efflux markedly increases brain penetration of ivermectin, resulting in prolonged activation of GABAergic and glutamate‐gated chloride channels and subsequent neurological toxicity. This mechanistic cascade provides a coherent explanation for the discrepancy between the favorable safety profile observed in controlled trials and the occurrence of severe neurotoxic events in genetically susceptible individuals or during pharmacological modulation of transporter activity.

Animal studies further elucidate the mechanistic basis of ivermectin neurotoxicity by demonstrating how species‐specific and breed‐dependent differences in BBB transporter function influence CNS exposure. In particular, certain canine breeds—most notably Collie‐type dogs—harbor inherited loss‐of‐function mutations in the ABCB1 (formerly MDR1) gene, resulting in deficient P‐glycoprotein–mediated efflux at the BBB and markedly increased brain penetration of ivermectin. Similar transporter deficiencies have been described in other species, including cats carrying ABCB1 deletions. In addition to genetic susceptibility, age‐dependent immaturity of the BBB in neonatal animals may reduce efflux capacity and increase central exposure. Experimental models further demonstrate that pharmacological inhibition or saturation of P‐glycoprotein can transiently compromise BBB protection even in otherwise resistant species. Thus, susceptibility does not reflect an inherently “incomplete” barrier structure, but rather quantitative differences in transporter expression, genetic integrity, developmental maturation, or pharmacological modulation that collectively determine the degree of ivermectin brain accumulation and subsequent neurotoxicity. Importantly, environmental and ecological studies extend the toxicological relevance of ivermectin beyond clinical medicine, highlighting sustained nontarget toxicity and ecosystem‐level effects that align with a One Health framework.

The COVID‐19 pandemic provided a unique and cautionary context in which ivermectin exposure patterns diverged markedly from approved therapeutic use. Off‐label administration was largely driven by early drug‐repurposing efforts following in vitro reports suggesting inhibition of SARS‐CoV‐2 replication through interference with importin‐α/β1–mediated nuclear transport, despite pharmacokinetic modeling indicating that the concentrations required for antiviral activity were unlikely to be achieved in humans at standard dosing. This translational gap, combined with widespread nonregulated treatment protocols, led to the use of heterogeneous ivermectin formulations, including oral human tablets administered at supratherapeutic or repeated prophylactic regimens and veterinary preparations such as 1.87% equine paste and 1% livestock solutions with substantially higher drug content. Clinical case series and poison center analyses consistently demonstrated that severe neurotoxicity was disproportionately associated with these high‐concentration veterinary products and atypical dosing schedules rather than with approved antiparasitic regimens. From a toxicological perspective, these events underscore that adverse outcomes during the pandemic were largely driven by altered pharmacokinetic exposure and formulation‐dependent bioavailability, highlighting the importance of distinguishing drug toxicity from exposure context when evaluating ivermectin safety.

Taken together, integrated human and animal data indicate that ivermectin neurotoxicity arises primarily from altered pharmacokinetic exposure rather than intrinsic loss of safety at therapeutic dosing. Across species, variability in ABCB1/P‐glycoprotein–mediated efflux, developmental maturity of the BBB, and pharmacological modulation of transporter activity collectively determine the degree of CNS penetration. Clinical reports during the COVID‐19 pandemic further emphasize that atypical formulations, supratherapeutic dosing, and prolonged exposure patterns substantially increase toxic risk. These observations support a unified toxicological framework in which formulation, exposure context, and individual susceptibility—rather than dose alone—govern the neurological safety profile of ivermectin.

## 8 Future Perspectives

Future research should prioritize a mechanistically grounded understanding of interindividual susceptibility to ivermectin toxicity, particularly through the lens of ABCB1/MDR1 P‐glycoprotein–mediated efflux at the BBB. Experimental and veterinary evidence has consistently demonstrated that transporter dysfunction profoundly alters CNS pharmacokinetics, as illustrated by ABCB1‐deficient mouse models, Collie‐type dogs carrying inherited MDR1 mutations, and cats with documented ABCB1 deletions, all of which exhibit markedly increased brain penetration and enhanced neurological toxicity following ivermectin exposure. These species‐specific observations provide a translational framework for interpreting rare but severe human toxicities associated with genetic transporter defects or pharmacological inhibition of P‐glycoprotein. Beyond genetic predisposition, future investigations should integrate pharmacogenomic screening, developmental BBB biology, and drug–drug interaction profiling to clarify how transporter expression, metabolic capacity, and exposure context collectively shape susceptibility across species.

Further mechanistic investigations are warranted to elucidate the determinants of ivermectin distribution within the CNS; however, the measurement of brain concentrations remains technically challenging and should be interpreted with caution. Quantitative data are largely limited to experimental animal models, including ABCB1‐deficient mice and susceptible canine breeds, where postmortem tissue analysis and controlled dosing studies have demonstrated marked increases in brain accumulation under conditions of impaired P‐glycoprotein–mediated efflux. Even in these settings, variability in sampling time, regional brain distribution, and lipid partitioning complicates direct pharmacokinetic comparisons. Importantly, reliable measurements of ivermectin concentrations in human brain tissue are essentially absent, and current knowledge relies primarily on indirect clinical observations, pharmacogenetic evidence, and rare postmortem toxicological reports. Consequently, extrapolation from animal brain concentration data to human neurological risk must remain cautious, emphasizing functional transporter activity and exposure context rather than absolute tissue concentration thresholds.

From a clinical standpoint, management of ivermectin intoxication remains primarily supportive, as no specific pharmacological antidote has been established for human use. Treatment focuses on airway protection, hemodynamic stabilization, seizure control, and monitoring for progressive CNS depression. Early gastrointestinal decontamination with activated charcoal may be considered in recent oral ingestions, although evidence is limited. Importantly, there is no approved antagonist capable of reversing ivermectin's potentiation of GABA‐gated chloride channels in humans. Experimental interventions in veterinary medicine have included the use of picrotoxin, a noncompetitive GABA_A receptor antagonist, and physostigmine, an acetylcholinesterase inhibitor, in dogs with ABCB1/MDR1 mutations; however, these approaches remain limited to controlled experimental settings and carry significant pro‐convulsant and cardiotoxic risks. Consequently, clinical recovery in most human cases reflects redistribution and elimination of the drug rather than pharmacological reversal.

At the public health level, the surge in ivermectin‐related poison center calls during the COVID‐19 pandemic underscored the consequences of unsupervised off‐label use and veterinary formulation exposure (Temple et al. 2021; Hoang et al. 2022). Increased hospitalization rates, intensive care admissions, and neurologic complications were disproportionately associated with supratherapeutic dosing and high‐concentration livestock products. These findings highlight the need for improved regulatory oversight, public education regarding formulation differences, and clearer communication of pharmacokinetic limitations identified in early translational studies (Caly et al. 2020; Guzzo et al. 2002).

Finally, environmental and ecological considerations further expand the toxicological relevance of ivermectin beyond clinical medicine. Aquatic microcosm studies have demonstrated sediment accumulation and severe invertebrate toxicity at concentrations corresponding to post‐treatment livestock excretion (Mesa et al. 2017). Similarly, chronic exposure experiments in zebrafish reveal concentration‐dependent behavioral and oxidative stress effects at sublethal levels (Domingues et al. 2016). These findings emphasize that ivermectin safety must be evaluated within a broader One Health framework encompassing human, veterinary, and environmental risk domains.

## Author Contributions

Serkan Yılmaz: conceptualization, investigation, data curation, formal analysis, and writing – original draft. Bayram Göktaş: conceptualization, investigation, data curation, formal analysis, and writing – original draft. İlker Ateş: methodology, visualization, writing – review and editing, and supervision. Mustafa Celik: methodology, visualization, writing – review and editing.

## Conflicts of Interest

The authors declare no conflicts of interest.

## Data Availability

The data that support the findings of this study are available from the corresponding author upon reasonable request.
